# Cholinergic blockade of neuroinflammation: from tissue to RNA regulators

**DOI:** 10.1042/NS20210035

**Published:** 2022-02-11

**Authors:** Tamara Zorbaz, Nimrod Madrer, Hermona Soreq

**Affiliations:** 1 The Edmond and Lily Safra Center of Brain Science and The Life Sciences Institute, The Hebrew University of Jerusalem, Jerusalem 9190401, Israel; 2 Biochemistry and Organic Analytical Chemistry Unit, The Institute of Medical Research and Occupational Health, Zagreb, Croatia

**Keywords:** acetylcholine, aging, cholinergic, microRNA, neuroinflammation, sex

## Abstract

Inflammatory stimuli and consequent pro-inflammatory immune responses may facilitate neurodegeneration and threaten survival following pathogen infection or trauma, but potential controllers preventing these risks are incompletely understood. Here, we argue that small RNA regulators of acetylcholine (ACh) signaling, including microRNAs (miRs) and transfer RNA fragments (tRFs) may tilt the balance between innate and adaptive immunity, avoid chronic inflammation and prevent the neuroinflammation-mediated exacerbation of many neurological diseases. While the restrictive permeability of the blood–brain barrier (BBB) protects the brain from peripheral immune events, this barrier can be disrupted by inflammation and is weakened with age. The consequently dysregulated balance between pro- and anti-inflammatory processes may modify the immune activities of brain microglia, astrocytes, perivascular macrophages, oligodendrocytes and dendritic cells, leading to neuronal damage. Notably, the vagus nerve mediates the peripheral cholinergic anti-inflammatory reflex and underlines the consistent control of body–brain inflammation by pro-inflammatory cytokines, which affect cholinergic functions; therefore, the disruption of this reflex can exacerbate cognitive impairments such as attention deficits and delirium. RNA regulators can contribute to re-balancing the cholinergic network and avoiding its chronic deterioration, and their activities may differ between men and women and/or wear off with age. This can lead to hypersensitivity of aged patients to inflammation and higher risks of neuroinflammation-driven cholinergic impairments such as delirium and dementia following COVID-19 infection. The age- and sex-driven differences in post-transcriptional RNA regulators of cholinergic elements may hence indicate new personalized therapeutic options for neuroinflammatory diseases.

## Introduction

Lack of spatiotemporal balance between immune system activation and suppression following pathogen infiltration or trauma and consequent inflammatory responses may threaten survival, initiating a search for potential controllers preventing these risks. In this context, genomic DNA elements appear at the top of the multilayer pyramid of gene expression. However, changes at the DNA level may lead to inherited risk and antagonistic pleiotropic responses in particular tissues or ages [1]. In contrast, specific protein regulators at the bottom of the pyramid may fail to block the multicomponent inflammatory state, whereas the interim layer that includes numerous short and long non-coding RNA (lncRNAs) transcripts may offer a solution to this daunting issue. Non-coding RNAs (ncRNAs) compose the great majority (≈98%) of transcripts of the human genome, may be dozens to tens of thousands nucleotides long, and are transcribed by a highly diverse class of genes [2]. The small non-coding RNAs (sncRNAs, less than 200 nucleotides in length) may be subclassified into microRNAs (miRs) [3], siRNAs, piRNAs, snoRNAs, snRNAs, and the newly rediscovered transfer RNA fragments (tRFs) [4], and the biological functions and role in disease pathogenesis of many of them is not well understood. Of those, both lncRNA (longer than 200 nucleotides) and sncRNAs are important but understudied regulators of cholinergic pathways and the inflammation processes.

SncRNAs in both the nervous and the immune systems may act as ‘negotiators’ operating between these two interacting compartments by targeting pertinent genes and co-modulating immune and neuronal processes [5–7] that jointly control the complex temporal dynamics of higher brain functions and affect cognition, neuroinflammation and behavioral processes regulated by the cholinergic network [8,9]. Brain neurons releasing acetylcholine (ACh) and participating in the cholinergic network are clustered in nuclei of brainstem areas, from where they project cholinergic inputs to cortical and subcortical structures [10] as well as from cholinergic interneurons distributed in cortical regions [11–14]. Other cholinergic neuron subtypes constitute a significant part of the peripheral nervous system, including the somatic and autonomic nervous system, and more specifically, preganglionic neurons of the sympathetic nervous system and both preganglionic and postganglionic nerves of the parasympathetic nervous system [15]. In addition to the nervous system, cholinergic genes handle ACh synthesis and release in epithelial cells, myocytes, apocrine, and immune cells [16].

SncRNAs are important in neuronal development and for maintaining homeostasis in adult neurons; for example, a specific subgroup of miRs operates by balancing neuronal and immune functions (NeurimmiR) [17], and changes in their levels may modulate neuronal or immune-related diseases. Moreover, miRs targeting cholinergic transcripts (CholinomiRs) are intimately associated with regular and pathological processes affecting cholinergic network functions, as they can act rapidly and effectively to down-regulate multiple targeted cholinergic transcripts carrying complementary sequence and can each operate by affecting many interconnected transcripts [18]. Notably, several CholinomiRs have been associated with cholinergic–immune system interactions [7]. Among other sncRNAs, rediscovered tRFs emerged as interesting new regulators that may function like miRs, in addition to other activities such is interaction with RNA-binding proteins [19,20]. This makes both miRs and tRFs particularly suitable for controlling the rapidly changing physiology of the cholinergic network under quiescent and acute neuroimmune conditions. Correspondingly, a ‘changing of the guards’ process of exchange in the levels of miRs and tRFs occurs in acute pathologies [19], alongside sex- and age-dependent differences in CholinomiRs [6,11]. Together, these features enable the use of small RNAs for further development of personalized therapy, and for the establishment of novel diagnostic tools.

## The cholinergic system’s coding genes

Cholinergic neurons express genes and proteins involved in ACh synthesis (choline acetyltransferase, ChAT; CHAT; 10q11.23) and within the same transcript, the packaging protein (vesicular ACh transporter, VAChT; SLC18A3; 10q11.23). Another key component is the high-affinity choline transporter (ChT; SLC5A7; 2q12.3) controlling re-uptake of the ACh precursor choline, which represents the rate-limiting step in ACh production [21]. Postsynaptic cholinergic cells express nicotinic (nAChR; α, β, γ, δ, ε subunits encoded by >15 genes) and muscarinic receptors (mAChR; M1–M5, 5 genes) and the major ACh hydrolyzing protein acetylcholinesterase, AChE (ACHE; 7q22.1) [22]. Another ACh hydrolyzing enzyme, butyrylcholinesterase (BCHE; 3q26.1) is mainly located extrasynaptically (e.g., in brain astrocytes or perisynaptic Schwann cells of the neuromuscular junction) [23–25].

Despite sharing the ability to synthesize ACh, cholinergic neurons show diverse phenotypes, transcript patterns and functions [10]. Correspondingly, the pre-transcriptional regulation of cholinergic genes involves diverse transcription factors and/or epigenetic modifications. During development, cholinergic differentiation from neural progenitors initiates with binding of the LIM homeodomain Isl1 transcriptional factors to specific cholinergic enhancers. In the developing forebrain, additional LIM proteins form hexamers with Isl1, e.g., the Isl1-Lhx8-hexamer which coordinates cholinergic neuron specification, whereas the Isl1-Lhx3-hexamers drive the spinal cord differentiation of cholinergic motor neurons [26–28]. Further, the Lhx6 transcription factor is expressed in a subset of brain cholinergic interneurons [29], and the Phox2b, Ret and Txl3 transcription factors drive sympathetic cholinergic neurons [30]. Neuregulin 1 (NRG1) and indirect agrin signals are involved in the differentiation of both neuromuscular synapses and cholinergic brain synapses [31]. Additionally, NRG1-activated Erb signaling leads to binding of GABP transcription factors to the N-box of several ACh receptor (AChR) promoters. Abnormalities of neuregulin signaling are involved in the pathogenesis of several human diseases, including schizophrenia and multiple sclerosis (MS) [31]. Interestingly, there are over 20 alternative splicing-derived isoforms of the NRG1 transcript and three more genes encode closely related proteins with incompletely elucidated functions [31].

To convey its signals, ACh binds to nicotinic or muscarinic AChRs. The nicotinic ionotropic channel receptors are largely mixed pentamers of mostly α (α2‒α7, α9, α10) and β (β2‒β4) subunits. In the brain, most abundant are the homopentameric α7 and the α4β2 and α3β4 heteromeric receptors [32,33]. Muscarinic receptors are G protein-coupled metabotropic receptors: their M1, M3, M5 members are coupled to Gq/11 proteins that up-regulate phospholipase C (PLC) and inositol triphosphate, increase intracellular Ca^2+^ concentrations and are mostly located in postsynaptic sites, whereas the M2 and M4 receptors are coupled to Gi/o proteins and may deactivate adenylate cyclase and activate K^+^ channels located at both the pre- and post-synaptic sites [32,34]. To add to this complexity, neuronal α7 nAChR may indirectly connect to G protein effects via the G protein-regulated inducer of neurite outgrowth 1 (Gprin1), empowering ACh to perform additional sustained activities [35]. On peripheral skeletal muscles, the most abundant are nAChRs [32], whereas autonomic ganglia, smooth muscles and other parasympathetic end organs, as well as epithelial, endothelial and immune cells harbor both mAChR and nAChR molecules. Specifically, brain mAChR and nAChR are widely distributed on cholinergic, glutamatergic and GABA-ergic neurons [36], as well as on microglia and astrocytes [32]. Altogether, several dozen genes compose this complex network and sncRNAs targeting them should hence operate in multiple tissues.

Immune cells express cholinergic proteins and cholinergic signaling plays substantial roles in immunological responses. Thus, both nicotinic and muscarinic AChRs appear on macrophages, lymphocytes (mostly CD4+ T cells) [19] and dendritic cells [16], which control innate and acquired immunity, and secrete both anti-inflammatory cytokines including IL-1ra, IL-4, IL-6, IL-10, IL-11 and IL-13, and pro-inflammatory cytokines such as TNFα, IL-1β, IL-6 and IL-8 [37,38]. Further, impaired regulation of part or all of the sncRNAs expressed in these cells could ignite chronic inflammatory or autoimmunity reactions [17]. Given this bidirectional complexity, identifying the molecular regulators of the reciprocal multimember interactions between cholinergic elements and inflammatory pathways is particularly challenging.

## Cholinergic control of anti-inflammatory and neuroimmune responses

**Figure 1:**
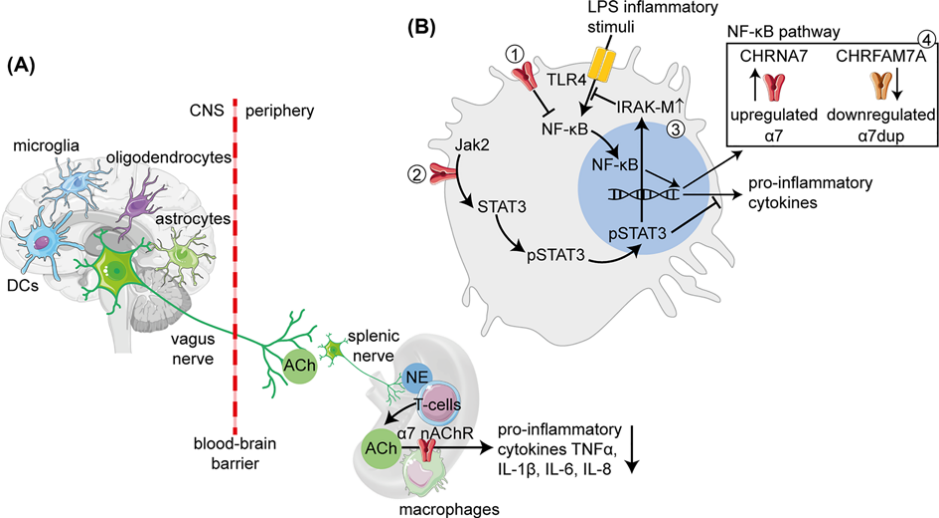
The cholinergic anti-inflammatory pathway (**A**) The proximity of cholinergic brain neurons to glia and the presence of AChRs on immune cells facilitate the cholinergic neuroimmune regulation. In peripheral tissues, communication of the ACh eliciting vagus nerve with the norepinephrine (NE) producing splenic nerve activates ACh production in T cells, where ACh initiates the cholinergic anti-inflammatory pathway by α7 nAChR activation and the suppression of pro-inflammatory cytokines secretion from splenic macrophages. (**B**) Molecular mechanisms involved in cholinergic anti-inflammatory actions involve the α7 nAChR-induced anti-inflammatory response that (1) inhibits the NF-κB pathway [43], (2) activates the JAK-STAT3 pathway (2) [41,45] or (3) up-regulates IRAK-M that attenuates TLR signaling (3) [46]. In addition, inflammatory stimuli up-regulate CHRNA7 and down-regulate its duplicated fused gene *CHRFAM7A* (4), thus promoting the anti-inflammatory actions [49].

The systemic inflammatory response is mediated via the vagus nerve (cranial nerve X), which provides brain-originated cholinergic regulation of inflammatory events both in the periphery and in the brain (Figure 1A). The afferent vagus nerve fibers sense pro-inflammatory cytokines in the periphery, whereas ACh brain-to-spleen signaling via the efferent vagus fibers limits the production of pro-inflammatory cytokines (TNFα, IL-1β, IL-6 and IL-8) in splenic macrophages [39–41]. More specifically, vagus-derived ACh regulates norepinephrine release from the splenic nerve, which accelerates ACh production in splenic T cells via adrenergic receptors [42]. ACh then activates the α7 nAChR on splenic macrophages [42,43], leading to declined pro-inflammatory cytokine production via different molecular mechanisms (Figure 1B). These mechanisms include blockade of the NF-κB pathway [43], activation of the JAK-STAT pathway [41,44,45] and up-regulation of IRAK-M that operates as a negative controller of the innate immunity Toll-like receptor (TLR) responses [46], all of which might include contributions of ncRNA regulators. Additionally, α7 nAChR-induced blockade of the NF-κB pathway inhibits macrophage secretion of HMGB1, a DNA chaperone that signals and contributes to inflammation by acting as a pro-inflammatory cytokine, inducing tissue-type plasminogen activator, stimulating the production of other pro-inflammatory cytokines (TNFα, IL-1β and IL-8) and promoting epithelial cell permeability [47]. Moreover, HMGB1’s redox state dictates the inflammatory or tissue regenerative activities, and its oxidized form sustains inflammation and promotes degeneration in muscular dystrophies [48]. Impaired ncRNA-modulated crosstalk between the cholinergic and immune systems is hence implicated in diverse pathological processes.

Balanced neuroinflammatory responses are essential for preserving healthy tissue and promoting tissue repair (neuroprotection); inversely, imbalanced, chronic neuroinflammation may initiate or propagate neuropathological events including neurodegenerative and mental diseases (Alzheimer’s disease (AD), Parkinson’s disease (PD), MS, schizophrenia (SCZ), bipolar disorder (BD) and others). Those drive neuroinflammatory events and/or alter brain immunoregulatory mechanisms in neurons, but also in microglia, astrocytes, perivascular macrophages (PVMs), oligodendrocytes or dendritic cells [50–53]. Likewise, traumatic brain injury, stroke or infections by neurotropic pathogens including COVID-19 may lead to long-term detrimental processes or lethal outcomes [19,54]. Further, the evolutionarily conserved physiological reaction to peripheral inflammatory insults spans brain-originated responses including fever, loss of appetite, lethargy, apathy, hypoactivity and reduced social interactions, that together can ensure energy preservation facilitating one’s ability to fight the infection, i.e., via sickness behavior [55,56]. This example of body–brain communication demonstrates the crosstalk between immune responses and cholinergic-related functions.

Inflammation may also disrupt surveillance by the blood–brain barrier (BBB) over brain entry of cytokines and other molecular invaders. Whereas tight BBB junctions between vascular epithelial cells are co-supported by surrounding pericytes and astrocytes [57], the circumventricular organs and choroid plexus are relatively permissible cytokine entry points into the brain [37], and choroid plexus macrophages produce cytokines such as IL-1β. Intriguingly, nicotine stimulation of nAChR in epithelial cells of the cerebral microvasculature can increase BBB permeability [58], whereas α7 nAChR activation in splenic macrophages reduces the inflammation-induced elevation of BBB permeability. Correspondingly, α7 nAChR-knockout (KO) mice show high TNFα and IL-1β levels and increased BBB permeability compared with wildtype mice, demonstrating BBB breakdown under pro-inflammatory environment [59]. Both BBB epithelial cells and brain glial cells express muscarinic and nicotinic AChRs, with species and brain region differences in their subunit composition [32], such that RNA regulators of these receptors may preserve BBB integrity while exerting a beneficial neuroimmune outcome within the brain. Hence, this calls for in-depth testing of the sncRNA controllers of the cholinergic network genes in the healthy and diseased brain. Correspondingly, recent reports of miR/tRF regulators of cholinergic neuronal and immune processes predict prognostic, diagnostic, and therapeutic potential for manipulating these sncRNAs in cholinergic-related diseases, affecting both brain function and the immune system in AD [60], PD [61], epilepsy [62], anxiety and depression-related disorders [63,64], mental disorders such as SCZ and BD [11], COVID-19 infection [54] and ischemic stroke [19]. Studying context-, time- and sex-dependent changes in RNA regulators can shed new light on cytokines, chemokines and reactive oxidative species produced by glia, epithelial or peripheral immune cells, the impact of which extends to learning and memory and tissue repair processes, among other consequences.

## Neuroinflammation-controlling cholinergic receptors, transporters and enzymes

The latitude of cholinergic-regulated immune response depends on AChR levels in splenic and brain immune cells and is primarily mediated via homopentamers of α7 nicotinic receptor subunits encoded by the CHRNA7 gene (α7 nAChR). The duplication of CHRNA7’s exons 5–10 and fusion to FAM7A’s exons A–E created the *CHRFAM7A* gene, which is highly expressed in the brain as well as in leukocytes, macrophages/monocytes, dendritic cells, microglia and astrocytes. CHRFAM7A yields two splice variants of the α7dup subunit that lack the signal peptide, the ACh-binding site and the entire N-terminal domain of the CHRNA7-derived α7 subunit, but can assemble with α7 subunits into pentamers. In primary monocytes and macrophages, CHRFAM7A reaches 200–1000-fold higher expression levels than CHRNA7, but LPS-induced inflammation of myeloid cell lines led to NF-κB pathway-mediated CHRNA7 increase and CHRFAM7A decline [49]. Also, cerebral ischemia elevates α7 nAChR levels in rat microglia and astrocytes [32], and the AChE inhibitor donepezil, which is also a ligand of α7 nAChR, up-regulates both CHRFAM7A and CHRNA7 [65]. Likewise, neuregulin up-regulates the α7 nAChR, thus reducing TNFα and IL-6 secretion in microglia [66]. Moreover, brain neurons express a heteromeric α7β2 nAChR receptor with yet unknown impact on the cholinergic anti-inflammatory pathway [67].

Stimulation of muscarinic receptors also modulates immune responses. Centrally active agonists of the M1 receptor and antagonists of the inhibitory presynaptic M2 receptor each enhances ACh release, attenuating serum levels of the pro-inflammatory TNFα by potentiating the efferent vagus nerve [68]. Moreover, the stimulation of muscarinic receptors on T cells leads to activation of immune and inflammatory responses (e.g., enhanced antigen-induced antibody response and the antigen-mediated T-cell proliferation) [69]. Further, the pan-muscarinic antagonist atropine elicits an anti-inflammatory effect by inhibiting the migration of leukocytes to the inflammation site [69]. Also, M1/M5 KO mice produce less antigen-specific antibodies and their spleen cells produce less TNFα and IL-6 cytokines than wildtype mice, whereas α7-signaling modifies T-cell differentiation and α7 KO mice produce more antigen-specific antibodies and more TNFα and IL-6 in the spleen than wildtype mice [16]. In addition, the central effect depends on microglial expression of mAChRs and differs in health and disease. Thus, the muscarinic agonist carbachol stimulates 10% of the brain’s microglia in healthy mice, but 60 and 25% of the microglia in stroke and AD mice models, respectively. In experimental animal models, neither MS nor glioma models showed such responses, indicating disease specificity of such mAChR elevation in activated microglia. Moreover, interferon (IFN)-γ treatment of cultured adult and neonatal microglia elevated carbachol-sensitive microglial populations by 60%, mostly due to CHRM3 up-regulation; no change in mAChRs expression was observed upon LPS exposure or anti-inflammatory IL-4 treatments. Further, M3 positive microglia display an activated phenotype, and mAChR stimulation reduced the IFN-γ-driven increase in phagocytic activity of microglia [70]. In a mouse stroke model, elevated M3 levels in brain microglia are crucial for better recovery of motor coordination and cognitive functions, and for smaller ischemic lesions in male but not female mice [71]. Moreover, acute M3 activation in male mice microglia led to elevated cytokines production, while chronic M3 activation attenuated LPS-induced pro-inflammatory cytokine release and sickness behavior [72]. This indicates sex-specific differences in cholinergic-immune regulation, as is detailed below.

## Cholinergic-immune processes and disease-related risks are sex- and age-dependent

Both sex and age are defining factors in immune system transcriptomics, and both immune response and cholinergic-related functions (e.g. cognition) decline with age [32]. Adult women mount stronger innate and adaptive immune responses as well as increased susceptibility to inflammatory and autoimmune diseases compared to men [73]. This sex-dimorphic innate immune response partially reflects distinct androgen and estrogen response elements (AREs, EREs) in the promoters of several innate immunity genes, as well as a direct effect of sex steroids on pretranscriptional immunity related transcripts and regulatory changes of X-chromosome-related genes. For example, the innate immune recognition of viral nucleic acids could be stronger in females where TLR7 escapes X-inactivation, leading to higher TLR7 levels. Further, exposure to TLR7 ligands elevates IFNα production in women- compared to men-derived blood cells [73].

Sex and age, along with different pathologies, are also defining factors of the cholinergic tone [6]. In the neuro–immune interface, ACh signaling is elevated under anxiety and functions as a modulator of the anti-inflammatory response [40]. For example, AD involves loss of cholinergic neurons in deep brain nuclei [74] and elevated neuroinflammatory events [75]. However, how the cholinergic–immune crosstalk mediates this characteristic cholinergic neuronal damage is still unknown. Moreover, AD is more prevalent in women than men, and atrophy of the language-related temporal cortex area is more pronounced in women [76,77]. This might reflect a stronger immune response of women in the periphery (i.e., higher CD4+ T lymphocytes, cytotoxic T cells and B cell counts, higher antibody production, activation of macrophages and phagocytic activity of immune cells) [73] as well as sex differences in brain immune cells—microglia [78–80], all of which could underlie these sex-related differences in AD progression. While the underlying mechanism explaining this sex-related misregulation in AD is incompletely understood, cortical tissues of individuals with schizophrenia and bipolar disorder showed pronounced sex-related differences in miRs targeting cholinergic and neurokine networks [11], corroborating these sex-related differences as caused/reflected by changes in sncRNA regulators of cholino-immune functions.

Microglial impairments have been demonstrated in various diseases [50], and ‘priming of microglia’ due to previous neuroinflammation or neuropathological conditions exacerbates microglial response with age under relatively small pro-inflammatory stimuli. Primed microglia are also more resistant to anti-inflammatory regulation, culminating in accelerated neuronal loss and neurodegenerative processes [50]. The mechanism by which inflammatory stimuli up-regulate M3 AChR in microglial and myeloid lineage cells may cause age-related change of the cholinergic–immune crosstalk. Also, microglia show sex dimorphism in many brain regions in terms of their number, morphology characteristics of ‘activation’ [81], as well as transcriptome and proteome profiles [82].

**Figure 2:**
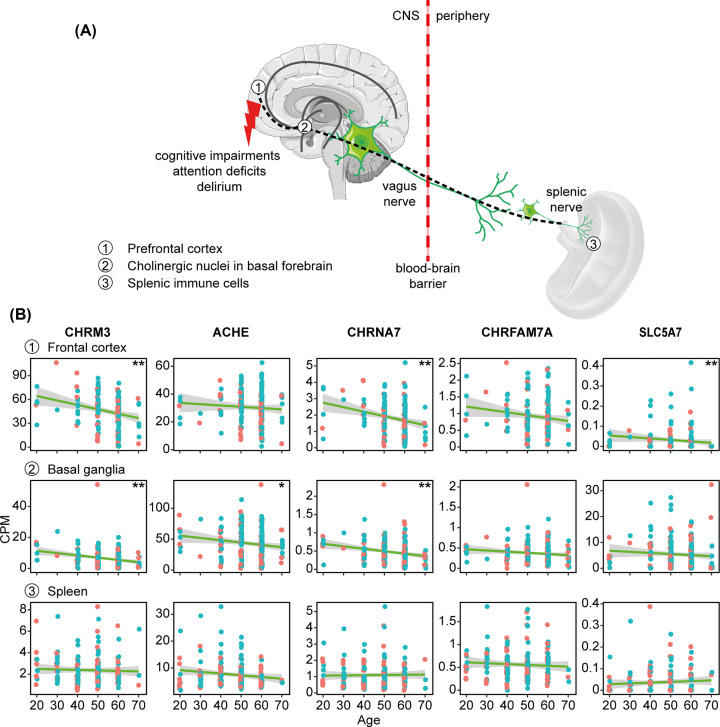
Cholinergic-neuroimmune modulation of attention deficits and impaired cognitive functioning reflect brain transcript changes (**A**) Higher cognitive functions such as attention are impaired in delirium and involve the prefrontal cortex (1), innervated by cholinergic neuron projections from cholinergic brain nuclei of the basal forebrain (2) and extending to the cholinergic anti-inflammatory brain–spleen circuit of the vagus nerve (3). (**B**) Age-dependent decline of CHRM3, ACHE, CHRNA7, CHRFAM7A, and SLC5A7 transcripts in the frontal cortex, basal ganglia and spleen. GTEx datasets of male and female transcript levels (blue and pink dots) change with age in both sexes. Note significant decreases in the frontal cortex for CHRM3 (r = −0.23, *P*=0.0081), CHRNA7 (r = −0.28, *P*=0.003), and SLC5A7 (r = −0.24; *P*=0.008), and in basal ganglia for CHRM3 (r = −0.22; *P*=0.0078), ACHE (r = −0.18; *P*=0.030) and CHRNA7 (r = −0.24; *P*=0.004). No significant change was observed in the spleen. Correlation is calculated using Spearman’s correlation test and green lines reflect linear fit. Adjusted *P*-values are FDR corrected. Subject numbers per the age groups of 20, 30, 40, 50, 60, 70 years were 5, 2, 13, 52, 85, 7 for frontal cortex; 7, 2, 21, 66, 94, 11 for basal ganglia; and 11, 19, 41, 60, 32, 3 for spleen.

AChR activation greatly depends on ACh production, where the rate-limiting step is choline re-uptake by the high-affinity ChT (SLC5A7), whereas ACh degradation is controlled by AChE. Changes and involvement of these proteins have been reported in AD neuro-inflammatory events. For example, neuronal AChE levels decrease, while glial BChE levels increase in AD brains [83]. Further, ChT levels associate with the amyloid precursor protein and participate in eliciting the AD cholinergic deficits [84]. Additionally, systemic inflammation impairs attention due to cytokine effects in the prefrontal cortex [85], and ChT levels relate to attention performance [21]. Moreover, a common genetic variant of ChT (IIe89Val) reduces the transporter activities by 50% and associates with cognitive deficits and attention-deficit/hyperactivity disorder (ADHD) syndrome [86]. The prefrontal cortex-basal forebrain-spleen cholinergic pathway contributes to neuroimmune event-triggered modulation of cognitive function such as attention (Figure 2A). Age-dependent changes in the frontal cortex, basal ganglia and spleen of transcript levels of α7 (CHRNA7), α7dup (CHRFAM7A), M3 (CHRM3), ChT (CHT) and AChE (ACHE) in the Genotype-Tissue Expression (GTEx) data (https://gtexportal.org/home/; accessed October 2021) may be causally involved (Figure 2B). Specifically, we identified age-related declines in CHRNA7 and CHRM3 in the frontal cortex and basal ganglia, SLC5A7 in the frontal cortex, as well as ACHE in the basal ganglia. Considering the lower than 1:1 glia:neuron ratio in the brain [87], and given that microglia compose up to ≈15% of total brain cells, the observed decline in cholinergic transcript levels may reflect altered cholinergic regulation of neuroimmune events occurring in microglia. Further, the M3 and α7 AChRs decline in the elderly brain may lead to reduced anti-inflammatory potential in aged individuals; thus, corroborating the increased risk of dementia in aged patients under prolonged anti-cholinergic medications [88].

## SncRNA modulators of the cholinergic–immune crosstalk

Both miRs and tRFs are post-transcriptional down-regulators of specific target genes that functionally interact with complementary recognition elements in the 3′ untranslated region (UTR) of the mRNAs of cholinergic target genes. Even though miRs are known to target many different mRNAs each, and particular mRNAs may be targeted by many miRs each, the specific involvement of particular miRs was shown to co-regulate targets of specific biological pathways. In the cholinergic pathway, certain miRs selectively target transcripts that control the synthesis of ACh (thus resulting in suppressed cholinergic tune), whereas other miRs target only the cholinesterases (yielding agonistic effect via preventing ACh breakdown) [6]. This suggests the existence of a mechanism by which notoriously redundant and non-specific processes might be precisely regulated. Therefore, miR/tRF-mediated suppressed translation [3,4] may control inflammation stimuli in a fast-acting and long-lasting manner as reflected, for example, by the age-declined transcript levels of brain AChRs. Since cholinergic signaling further involves transcripts that encode cytokines and other inflammation-related proteins, global changes in CholinomiRs may modulate pathological inflammation- and anxiety-related processes, brain and cardiac diseases, neurodegenerative processes and pain [89]. In particular, miR controllers of ACh production and destruction may modulate both the neuronal and immune functions of ACh [17,90], modifying neuroinflammation, whereas miRs targeting AChE and BChE could exert anti-inflammatory impact by suppressing ACh degradation and increasing ACh-mediated blockade of pro-inflammatory pathways. Additionally, numerous miRs associated with inflammation and anxiety are predicted to target cholinergic genes (e.g., AChE, VAChT) [89].

While miRs are important cholinergic signaling regulators [5], other sncRNA regulators of the cholinergic network, such as tRFs are also elicited under neuroinflammation. Those can lead to modified levels of multiple cholinergic genes and provide consistent sex-related surveillance of the cholinergic–neuroimmune regulation. For example, miR regulators of cholinergic signaling showed differential expression in the brain of male and female patients with mental diseases [11], male and female microglia from both healthy and tauopathy mice express different miR sets in a sex hormone-influenced manner [91] and tRF profiles are largely modified in nucleated blood cells from post-stroke patients [19].

Relevant examples include the inflammation-promoting miR-155, the inflammation-suppressing miR-146a, miR-124 and miR-21, and let-7 family members that may either promote or inhibit the inflammatory response [92,93]. Notably, miR-146a acts as a negative feedback regulator of NF-κB signaling by targeting its interleukin-1 receptor-associated kinase 1 (IRAK1) and TNF receptor-associated factor 6 (TRAF6) components, as well as signal transducer and activator of transcription 1 protein (STAT1), interferon regulatory factor 5 (IRF5) and complement factor H (CFH) [93]. Consequently, IRF5 targeting of STAT1 and IRAK1 provides synergistic anti-inflammatory effects with cholinergic α7 nAChR activation. Further, α7 nAChR-mediated up-regulation of interleukin-1 receptor-associated kinase 3 (IRAK-M) inhibits NF-κB signaling by preventing phosphorylation and dissociation of IRAK1, interrupting its activation via NF-κB-dependent proinflammatory signaling [46,94]. Moreover, IRAK1 is encoded by the X-chromosome, such that α7 nAChR-activation might be less effective in females with X-inactivation escape of IRAK1, where higher expression of miR-146a could be the key regulator of possible IRAK1-induced inflammation. Also, the basal levels of another miR-146a target, IRF5, are higher in immune plasmacytoid dendritic cells (pDCs) of females than males due to its ERα-mediated expression [73].

In macrophages, miR-21 mediates anti-inflammatory effects by decreasing TNFα secretion [93], whereas let-7 family miRs modulate neuroinflammatory processes by targeting signal transducer and activator of transcription 3 (STAT3) and promoting the polarization of macrophages into their anti-inflammatory profile [92]. Let-7 promotes the activation of microglia and macrophages by serving as a damage-associated molecular patterns (DAMPs) agent and an alert signal for innate immune activation via TLR7, a chromosome X-transcribed gene which may induce more pronounced inflammation in females with X-inactivation escape, calling to investigate the sex-dependent differences in let-7 levels. Moreover, some members of let-7 are predicted to target AChE and BChE [89].

Numerous neuroimmune-regulating miRs, including miR-124 and miR-132, function within both the nervous and the immune systems and may act as ‘negotiators’ between these two interacting compartments [17]. Microglia-expressed miR-124 is up-regulated under α7-nAChR activation in macrophages and reduces inflammation by down-regulating TNFα, MHC-II and reactive oxygen species. By targeting STAT3 and TNFα converting enzyme (TACE), miR-124 leads to decreased secretion of pro-inflammatory cytokines (IL-6, TNFα) [45] and other pro-inflammation-related transcripts. Human miR-124 predictably targets the alternatively spliced AChE variants AChE-S, AChE-R [89] and the hippocampal Lhx2 transcription factor affecting axonal genesis [95]. In comparison, miR-132 operates as a functional regulator of the brain-to-body resolution of inflammation by targeting both the AChE-R and AChE-S transcripts and its elevation is hence associated with inflammation and anxiety [7], cardiac diseases [96] and neurodegeneration [89,97], whereas its suppression controls liver fattening [98]. Activated M1 and M3 receptors may potentiate miR-132 transcription via elevating intracellular calcium levels and exacerbating its binding to the calcium response element in the miR-132 promoter [6]. miR-608 is another primate-specific miR shown to target AChE, and its reduced targeting efficacy in case of a common 3′-UTR AChE SNP associates with massively elevated brain AChE levels, trait anxiety and inflammation, prefrontal activity under exposure to stressful insults and blood pressure [64,99,100].

Several miRs target mRNAs of the human nAChR subunits (miR-491 and a2, α10, β2; miR-494 and α3, α4, α7; miR-542-3p and β2; miR-766 and α4, α5; miR-668 and α2, α4, β2; miR-7a with α9, α10, β2, β4). Recognition elements in nAChR 3′-UTRs of miR-494, miR-542-3p, and miR-667/766 are conserved in rodents and humans, as well as in 3′-UTRs of ChT, AChE and BChE, and nAChR subunit chaperone proteins [101]. Moreover, nicotine down-regulates several miRs that target nAChR subunits, as well as miR-542-3p that correlated with β2 up-regulation, while not affecting the expression of miR-494 and α4 [101]. The human, but not murine α7 nAChR subunit is targeted by miR-211, which was implicated in the regulation of epileptiform activity [102]. Likewise, miR-128 predictably targets CHRM2 mRNA [103] and miR-107 targets CHRM1 whose levels are elevated in some individuals with schizophrenia [104], and miR-30e inhibits CHRM3 [105]. Further, NF-κB mediated M3 activation down-regulates miR-376b-5p, which promotes myocardial ischemia, possibly by inhibiting brain-derived neurotrophic factor (BDNF) expression [106]. Interestingly, miR-129-5p is involved in cortical neurogenesis by targeting the epidermal growth factor receptor (EGFR) and is regulated by choline availability [107].

Intriguingly, genes that are crucial for controlling the prefrontal cortex-basal forebrain-spleen cholinergic signaling axis are predictably targeted by miRs implicated in regulating immune-driven attention deficits (Figure 2A). Several of those interactions have been found using the mirDIP engine and some were validated experimentally. Notably, the inflammation and cognition-regulating genes *CHRNA7, ACHE, CHRFAM7A* and *SLC5A7* attract considerably more miRs than CHRM3 (Table 1).

**Table 1:** miRs carrying complementary regions to α7 (CHRNA7), α7dup (CHRFAM7A) nAChR subunits, M3 AChR (CHRM3), AChE (ACHE) and ChT (SLC5A7)

Target	CHRM3	ACHE	CHRNA7		CHRFAM7A	SLC5A7
**Validated miRs** ^1^	miR-30e	miR-132	miR-211		n.a.	n.a.
**Predicted human miRs** ^2^	miR-6776-5p miR-103a-1-5p miR-6880-3p miR-6803-3p miR-9718 miR-6786-3p	miR-125b-5p miR-125a-5p miR-423-5p miR-920 miR-483-5p miR-744-5p miR-7111-5p miR-658 miR-6894-5p miR-187-3p miR-25-5p miR-1228-5p miR-128-1-5p miR-4743-5p miR-6765-5p miR-6781-5p	miR-4692 miR-4514 miR-4684-5p miR-4673 miR-4680-5p miR-3174 miR-1291 miR-3925-3p miR-219b-3p miR-1587 miR-4695-3p miR-4741 miR-8055 miR-4675 miR-329-5p miR-6757-3p	miR-4649-5p miR-6744-5p miR-454-5p miR-4486 miR-6729-5p miR-6822-5p miR-4467 miR-1273h-3p miR-1537-3p miR-4523 miR-6720-3p miR-10400-5p miR-3193 miR-3648 miR-6084 miR-4785	miR-4692 miR-4514 miR-4263 miR-1587 miR-1291 miR-4741 miR-4690-3p miR-3174 miR-4675 miR-4680-5p miR-219b-3p miR-1298-3p miR-1273h-3p miR-662 miR-105-3p miR-6775-3p	miR-4659a-3p miR-4659b-3p miR-4760-3p miR-3149 miR-3118 miR-491-3p miR-376a-5p miR-5589-3p miR-1207-3p miR-363-5p miR-744-3p miR-3934-3p miR-4453 miR-3134 miR-6808-3p miR-6841-3p
		miR-4281 miR-1199-3p miR-10392-5p	miR-105-3p miR-1298-3p miR-6893-3p	miR-6722-5p miR-4783-5p miR-6078		miR-7705

1 Validated experimentally: miR-30e [105], miR-132 [7], miR-211 [102].

2 Predicted by unidirectional search in the mirDIP database that identifies the top 1% miRs by combined confidence scores.

Recently, tRFs are rapidly being recognized as regulators in a variety of biological processes and as being essential for the maintenance of tRNA metabolism and tRNA processing enzymes. Furthermore, modified tRF levels are being implicated in different pathologies, such as stress injuries, cancer, neurodevelopmental and neurodegenerative disorders [108,109]. Some tRFs may be yet more specific than miRNAs in their suppression capacity of mRNAs carrying complementary sequences, which might be due to their context-dependent expression in certain tissues and systems. For example, 5′-halves of tRNA (5′-tRFs) are more efficiently expressed in immune system organs compared with other tissues, and thus have been deemed as systemic immune signaling sncRNAs involved in the homeostasis of immune functions. Correspondingly, their blood levels can reflect certain pathophysiological events [110]. Respiratory syncytial virus infection induces abundant production of ≈30 nucleotide long 5′-half tRFs [111]. Moreover, some 5′-half tRFs are sex hormone-dependent (i.e., SHOT-RNAs; sex hormone-dependent tRNA-derived RNAs) and are highly expressed in ER-positive breast cancer and AR-positive prostate cancer cell lines [112]. Further, elevated levels of a specific 5′-half tRF (tRNAHisGUG) under infection and can activate the innate immune receptor TLR7 [113], considered to participate in the sex differences of the immune system.

Further, tRF can be associated with disease phases as elevated tRF levels were observed in nucleated blood cells of post-stroke patients [19] and in the pre-seizure period of individuals with epilepsy [114]. Intriguingly, the post-stroke response involved reciprocal changes between miRs and tRFs targeting cholinergic genes, with general up-regulation of tRF species [19], reminiscent of the sncRNA remodeling in T-cell activation and immune responses [115].

Besides sncRNAs, lncRNAs are also involved in post-transcriptional regulation of gene expression via ‘sponging’ of miRs and preventing them exerting any action on target mRNAs. Two inflammation-related lncRNAs, DANCR and NEAT1, are involved in regulating the inflammatory status of cholinergic neurons and in the susceptibility for COVID-19 infection. Moreover, both these lncRNAs showed sex- and age-dependent changes [54]. Similarly, several long non-coding circular RNAs (circRNAs) contribute to the immune responses [116] and are highly abundant in the brain [117] where they accumulate with age [118] but not in neurodegenerative disease brains [119]. Therefore, the up-regulation of circRNAs associates with changes in ncRNA-mediated post-transcriptional regulation that might reflect the long-term changes in cholinergic–immune functions in aged individuals.

In conclusion, numerous diseases reveal unique miR ‘signatures’ that can be exploited as diagnostic and prognostic biomarkers [120], and neurological disorders with neuroinflammatory context such as AD, epilepsy, PD, glioblastoma, MS and myasthenia gravis (MG) are accompanied by modified levels and/or properties of numerous miRs. Examples include miR-132, miR-124, and miR-155 in MS and MG [121] and decline of miR-9, miR-132, miR-212 and up-regulation of the AChE-targeted miR-125-b and miR-146 in AD [17]. However, this picture is far from complete as miRs may be replaced by tRFs reflecting a ‘changing of the guards’ process under acute situations. The predicted links between miRs and tRFs and neuroinflammatory events await further studies.

## Future prospects for elucidating post-COVID attention deficits and delirium mechanisms

Cholinergic signals consistently supervise attention and cognition processes via both the nicotinic and muscarinic ACh pathways [34], and diverse disorders of attention and cognitive control involve altered cholinergic signaling [36]. Among those, delirium is characterized as an acute confusion state marked by global impairments in attention and cognition, and cholinergic deficiency in the brain is a major pathophysiological process in delirium [122]. Thus, long-term use of anticholinergic drugs and cholinergic deficiency are recognized as likely contributing features to all causes of delirium, with a pivotal contribution of antimuscarinic effects in dementia and delirium-contributing processes [123]. Reciprocally, neuroinflammation is a risk factor in ADHD [124], and pro-cholinergic processes and nicotinic stimulation are implicated in ameliorating ADHD symptoms [125]. That impaired cholinergic functioning associates with both the age- and sex-related risks of delirium raises considerable concerns for post-COVID-19 long-term neuroimmune, cholinergic and cognitive changes. Recent reports confirmed increases in the cholinergic transcripts capable of controlling inflammation in lung cells infected by SARS-CoV-2. Moreover, those changes negatively correlated with modified profiles of the lncRNAs DANCR and NEAT1, which could promote the susceptibility to more severe outcomes of COVID-19 infection [54]. Also, while systemic inflammation caused by COVID-19 exposure activates peripheral cholinergic anti-inflammatory mechanisms, it further modulates brain activities with direct involvement in cholinergic brain functions. Supporting this concept, ∼70% of intensive care unit COVID-19 patients presented delirium symptoms that were likely connected to the underlying anti-cholinergic mechanisms [123], and associated with neuropsychological impairments. These symptoms lasted several months after discharge, based on data collected from a single-center cohort that supported previous findings of higher delirium incidence in critically ill patients with COVID-19 [126]. Further, COVID-19-induced delirium was correlated to the higher inflammatory response and 70% of COVID-19 patients with delirium were females [126]. This adds up to the consistent reports of increased risk of dementia in aged patients under long-term anticholinergic medications [88]. Furthermore, COVID-19-related changes in ncRNAs and transcription factors reflect increased risks of developing lung inflammation and cholinergic neurons demise [54], and elevated risk of severe COVID-19 was linked to the genetic OAS1 variant that associates with elevated risk of AD [127]. Together, these reports highlight the multileveled contributions of age, sex and genetic background in nervous system disorders connected to cholinergic and neuroimmune changes.

**Figure 3:**
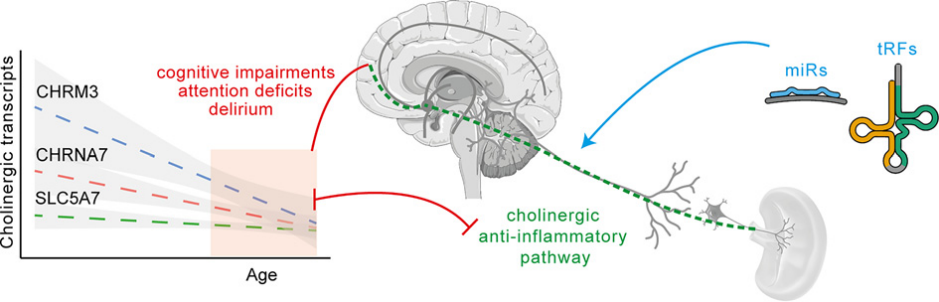
Role of sncRNAs (miRs, tRFs) in the regulation of cholinergic anti-inflammatory pathway Age-related decline of cholinergic transcripts in the prefrontal cortex might contribute to the susceptibility to age-related and/or disease-originated cognitive function deficits, whereas regulation at the level of sncRNAs might be a plausible future therapeutic intervention.

At the genomic level, identifying SNPs that might imbalance the neuroprotective features of cholinergic signaling may serve as a warning for drastic neuroinflammatory events, especially in the prefrontal cortex. Prefrontal cortical changes in cholinergic gene expression might be key contributors to balanced neuroprotection-neurodegeneration, which is causally involved in cholinergic functions such as attention; and malfunctioning of this balance is one of the characteristics of delirium. Further, the prefrontal cortex shows the highest degree of age-related atrophy, leading to larger risks of neurobehavioral and neuropsychological disorders [128]. Supervised by sncRNAs known to participate in controlling specific cholino-immune events, CHRNA7, CHRM3 and SLC5A7 and other cholinergic genes show age- and sex-related differences. Future studies of cholinergic-related post-COVID long-term neurological impairments signaled by delirium should hence be focused on such sncRNAs (Figure 3) and may reveal plausible therapeutic targets for disease and age-related dementia, which we suspect may significantly increase in the upcoming years.

## Data Availability

This analysis contains transcriptomics data of healthy individuals obtained using the GTEx database available online (https://gtexportal.org/home/).

## Open Access

Open access for this article was enabled by the participation of The Hebrew University of Jerusalem in an all-inclusive *Read & Publish* pilot with Portland Press and the Biochemical Society under a transformative agreement with *MALMAD*.

## Abbreviations

ACh: acetylcholine
AChE: acetylcholinesterase (ACHE)
AChR: ACh receptor
AD: Alzheimer’s disease
ADHD: attention-deficit/hyperactivity disorder
BBB: blood–brain barrier
BChE: butyrylcholinesterase (BCHE)
ChAT: choline acetyltransferase
CHRFAM7A: duplicated and fused CHRNA7 gene
CHRM3: M3 mAChR
CHRNA7: α7 nAChR
ChT: choline transporter (SLC5A7)
circRNA: circular RNA
DANCR: differentiation antagonizing non-protein coding RNA
IL: interleukin
IRAK1/-M: interleukin-1 receptor-associated kinase 1/3
IRF5: interferon regulatory factor 5
KO: knockout
lncRNA: long non-coding RNA
LPS: lipopolysaccharide
mAChR: muscarinic AChR
MG: myasthenia gravis
miR: microRNA
MS: multiple sclerosis
nAChR: nicotinic AChR
NEAT1: nuclear paraspeckle assembly transcript 1
ncRNA: non-coding RNA
NRG1: neuregulin 1
OAS1: 2',5'-oligoadenylate synthetase 1
PD: Parkinson’s disease
pDCs: plasmacytoid dendritic cells
sncRNA: small non-coding RNA
SNP: single-nucleotide polymorphism
STAT1/3: signal transducer and activator of transcription 1/3
TACE: TNFα converting enzyme
TLR: Toll-like receptor
TNFα: tumor necrosis factor-α
tRF: transfer RNA fragment
UTR: untranslated region
VAChT: vesicular ACh transporter (SLC18A3)
